# *Lactobacillus rhamnosus* 069 and *Lactobacillus brevis* 031: Unraveling Strain-Specific Pathways for Modulating Lipid Metabolism and Attenuating High-Fat-Diet-Induced Obesity in Mice

**DOI:** 10.1021/acsomega.4c02514

**Published:** 2024-05-16

**Authors:** Pin-Yu Ho, Ya-Chun Chou, Yen-Chun Koh, Wei-Sheng Lin, Wei-Jen Chen, Ai-Lun Tseng, Chiau-Ling Gung, Yu-Shan Wei, Min-Hsiung Pan

**Affiliations:** † Institute of Food Science and Technology, National Taiwan University, No. 1, Sec. 4, Roosevelt Road, Taipei 10617, Taiwan, ROC; ‡ Department of Food Science, National Quemoy University, Quemoy County 89250, Taiwan, ROC; § Syngen Biotech Co., Ltd., Building A, No. 154, Kaiyuan Rd., Sinying, Tainan 73055, Taiwan; ∥ Department of Public Health, China Medical University, 91, Hsueh-Shih Road, Taichung 40402, Taiwan, ROC; ⊥ Department of Food Nutrition and Health Biotechnology, Asia University, 500, Lioufeng Rd., Wufeng, Taichung 41354, Taiwan, ROC

## Abstract

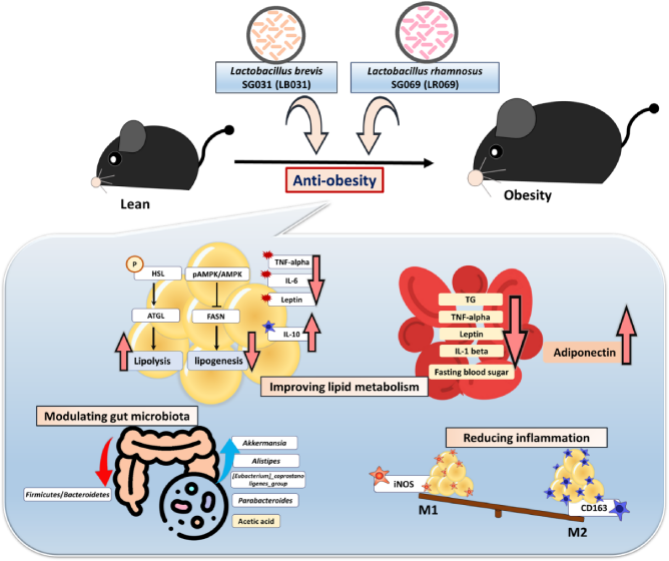

Obesity is a global health crisis, marked by excessive fat in tissues that function as immune organs, linked to microbiota dysregulation and adipose inflammation. Investigating the effects of *Lactobacillus rhamnosus* SG069 (LR069) and *Lactobacillus brevis* SG031 (LB031) on obesity and lipid metabolism, this research highlights adipose tissue’s critical immune-metabolic role and the probiotics’ potential against diet-induced obesity. Mice fed a high-fat diet were treated with either LR069 or LB031 for 12 weeks. Administration of LB031 boosted lipid metabolism, indicated by higher AMP-activated protein kinase (AMPK) and acetyl-CoA carboxylase (ACC) phosphorylation, and increased the M2/M1 macrophage ratio, indicating LB031’s anti-inflammatory effect. Meanwhile, LR069 administration not only led to significant weight loss by enhancing lipolysis which evidenced by increased phosphorylation of hormone-sensitive lipase (HSL) and adipose triglyceride lipase (ATGL) but also elevated *Akkermansia* and fecal acetic acid levels, showing the gut microbiota’s pivotal role in its antiobesity effects. LR069 and LB031 exhibit distinct effects on lipid metabolism and obesity, underscoring their potential for precise interventions. This research elucidates the unique impacts of these strains on metabolic health and highlights the intricate relationship between gut microbiota and obesity, advancing our knowledge of probiotics’ therapeutic potential.

## 1 Introduction

Obesity, characterized by excessive body fat accumulation through fat cell enlargement (hypertrophy) and increased fat cell numbers (hyperplasia),^1^ is associated with severe medical conditions such as cardiovascular disease, hypertension, dyslipidemia, type 2 diabetes, and cancer, posing significant health risks.^2^ Consequently, it has emerged as a major public health concern.^3^ White adipose tissue (WAT), the primary fat-storing depot and the largest endocrine organ, releases adipokines and cytokines systemically, contributing to chronic low-grade inflammation, termed metaflammation, and altered metabolism.^4^ Leptin, elevated in obesity, plays a crucial role in regulating the inflammatory response, influencing development, proliferation, maturation, and activation. Notably, leptin promotes the synthesis of pro-inflammatory cytokines.^5,6^ The induction of leptin expression signals a molecular mechanism linked to inflammatory activity, as its levels increase in adipose tissue due to inflammatory conditions such as hypoxia and inflammatory mediators commonly experienced in expanding adipose depots.^7^ Adipose tissue, a complex organ with diverse functions in energy storage, metabolic regulation, and neuroendocrine and immune functions, is now recognized as an immune organ at the intersection of metabolism and immunity.^8^ Macrophages within adipose tissue can polarize into M1 and M2 states, where M1 macrophages produce pro-inflammatory cytokines, contributing to inflammation, while M2 macrophages have an anti-inflammatory effect.^9^ The secretion of specific cytokines and chemokines from macrophages can modify the microenvironment, bridging innate and adaptive immune responses.^10^ Consequently, there is a growing interest in elucidating the relationship between inflammation and obesity-related metabolic diseases and in exploring the potential to ameliorate metabolic diseases through anti-inflammatory actions and enhanced M2 polarization.^11^

In recent years, the Westernization of dietary patterns has not only led to increased calorie consumption but has also fostered long-term habits of high-fat, low-fiber diets that have disrupted the intestinal microenvironment.^12^ These dietary shifts have triggered alterations in the composition of the human gut microbiota, with a marked decrease in cellulose-digesting intestinal bacteria and an increase in those capable of metabolizing the byproducts of high-fat diets.^13^ Research has revealed that obese individuals exhibit significant changes in the ratio of *Firmicutes* to *Bacteroidetes* within their gut microbiota compared to healthy individuals.^14^ These shifts in the bacterial composition may have profound implications for nutrient absorption within the intestines and the body’s overall energy metabolism equilibrium.^15^ As a result, investigating methods to address these changes in the context of obesity has become a prominent research focus.^16^ According to the definition by WHO, probiotics are living microorganisms found in certain foods; these include bacteria that normally exist in the human intestine and can improve the health of the host.^17^ Probiotic intervention is an important tool for improving the health of hosts.^18^ Probiotics are increasingly recognized for their potential in the prevention and treatment of obesity. Therefore, it is necessary to carefully screen lactic acid bacteria with antiobesity effects and explore their underlying mechanisms. This study aimed to investigate whether *Lactobacillus rhamnosus* SG069 (LR069) and *Lactobacillus brevis* SG031 (LB031) can reduce high-fat-diet-induced obesity in mice and elucidate the related mechanisms. We analyzed the effects of these lactic acid bacteria on intestinal bacteria and short-chain fatty acid composition and explored their effects on the inflammatory response and lipid accumulation caused by high-fat diet.

## 2 Experimental Section

### 2.1 Materials

Antibodies against p-acetyl-CoA carboxylase (p-ACC) (Ser79) and adipose triglyceride lipase (ATGL) were from Santa Cruz Biotechnology, Inc. (Dallas, TX). Antibodies against glyceraldehyde 3-phosphate dehydrogenase (GAPDH) were purchased from Abcam PLC (Cambridge, UK). Antibodies against phosphorylated (*p*-) and unphosphorylated hormone-sensitive lipase (HSL), Ac-CC, AMP-activated protein kinase (AMPK), and p-AMPK were purchased from Cell Signal Technology (Beverly, MA). Fatty acid synthase (FASN) was from Proteintech (Rosemont, IL). The Bio-Rad protein assay dye reagent for protein quantification was obtained from Bio-Rad Laboratories (Munich, Germany). Xylenes and hematoxylin and eosin (H&E) for staining were purchased from Leica Biosystems. *Lactobacillus brevis* SG031 (LB031) originates from fermented vegetables, while *Lactobacillus rhamnosus* SG069 (LR069) is sourced from the intestinal tract of healthy adults. Sample preparation involved centrifugation of the supernatant following liquid culture of the strains, followed by freeze-drying of viable bacteria to prepare test samples. The powders of LR069 and LB031 were supplied by Syngen Biotech Co. Ltd., Taiwan (R.O.C.).

### 2.2 Animals and Experimental Design

Thirty-two C57BL/6 male mice (20 ± 2 g, 4 weeks old) were purchased from the BioLASCO Taiwan Co. Ltd., Taipei City, Taiwan (R.O.C.) and were housed in an environmentally controlled room (temperature: 23 ± 2 °C, 50% relative humidity, conventional 12-h light/dark cycle) with food and water ad libitum. All experimental procedures involving animals were performed in accordance with the Laboratory Animal Care Guidelines of the Taiwan Ministry of Agriculture, and the protocol was approved by the Institutional Animal Care and Use Committee (IACUC) of National Taiwan University (Approval Number: NTU-110-EL-00014), ensuring ethics compliance without encountering unexpected or unusually high security issues. After 1 week of normal diet, the animals were randomly assigned into four groups (*n* = 8 per group) with different diets for 12 weeks: (1) ND group: normal diet (total calories 3.36 kcal/g, 13.4% calories in fat), oral gavage with double-distilled water every day; (2) HFD group: high-fat diet group (total calories 4.58 kcal/g, 50.4% calories in fat), oral gavage with double-distilled water every day; (3) HFD + LR069 (*Lactobacillus rhamnosus* SG069): HFD plus LR069 at 5 × 10^8^ CFU/mL/day. (4) HFD + LB031 (*Lactobacillus brevis* SG031): HFD plus LB031 at 5 × 10^8^ CFU/mL/day.

### 2.3 Dosage Information

The dosage regimen of 5 × 10^8^ CFU/mL/day used in this study was established based on an extensive review of both human and animal studies. This review ensured that the dosage aligns with the metabolic rate differences between species, thereby ensuring relevance and safety for translational applications. For this study, while specific lower and higher dose testing was not conducted, the chosen dosage is robustly supported by the scientific literature. Probiotic doses ranging from 1 × 10^8^ to 10^11^ CFU per day have been shown to effectively balance efficacy and safety across various experimental models.^19−22^ The chosen dosage of 5 × 10^8^ CFU/mL/day strikes a balance between demonstrating efficacy and maintaining practical considerations such as cost-effectiveness and feasibility of administration. This approach is designed to optimize potential benefits while minimizing the risks of excessive dosages that might not yield proportional improvements in health outcomes or could induce dysbiosis and other adverse effects.

### 2.4 Serum Biochemical Index

Blood samples were obtained and centrifuged at 4 °C at 1500 × g for 20 min to isolate the sera, which were then stored at −80 °C until analysis. The biochemical analysis was carried out in collaboration with the National Laboratory Animal Center (NLAC) located in Taipei, Taiwan, utilizing a 7080 Biochemical Analyzer manufactured by Hitachi in Tokyo, Japan, following the manufacturer’s guidelines. The serum specimens were analyzed, determining ALT (alanine transaminase), AST (aspartate aminotransferase), TC (total cholesterol), triacylglycerol (TG), HDL (high-density lipoprotein), and LDL (low-density lipoprotein) levels.

### 2.5 Adipocytokines and Liver Triglyceride Levels

The adipocytokines levels and triglyceride levels were measured using the relevant ELISA kits according to the manufacturer’s instructions. Liver tissues were analyzed for triglycerides (Cat. 10010303, Cayman). The serum was analyzed for adiponectin (ADIPOQ; Cat. ab108785; Abcam), leptin (Cat. EZML-82K, Millipore Corporation), and tumor necrosis factor-alpha (TNF-α; Cat. 88−7324; Thermo Fisher Scientific). The perigonadal adipose tissue was analyzed for leptin (Cat. ADI-900−019A; Enzo Life Sciences)^23^ and IL-10 (Cat. E-UNEL-M0057; Elabscience).

### 2.6 Histological and Immunofluorescence Staining

Liver and adipose tissues were sampled and fixed in 10% formalin buffer, followed by dehydration and paraffinization. Formalin-fixed paraffin-embedded tissues were cut to 3−5 μm in thickness, deparaffinized in xylene, and rehydrated in ethanol/water before staining with hematoxylin−eosin. Images were captured with an Olympus BX51 microscope, and the size distribution of subcutaneous WAT adipocytes was determined by using ImageJ software (Rasband, W.S., ImageJ, U.S. National Institutes of Health, Bethesda, MD). Xylenes and hematoxylin and eosin (H&E) stain were acquired from Surgipath (Peterborough, UK). For immunofluorescence staining, perigonadal fat tissues were stained with iNOS, CD163, and β-actin antibodies from Santa Cruz Biotechnology, Inc. (Dallas, TX), followed by secondary antibodies. The mouse β-actin monoclonal antibody and DAPI were obtained from Sigma Chemical Co. (St. Louis, MO).^24^

### 2.7 Western Blotting

The protein extraction process involved homogenizing tissue samples with a Polytron tissue homogenizer for 10 s. A lysis buffer containing a protease inhibitor cocktail was then added, and the mixture was incubated for 1 h at 4 °C. After centrifugation at 10,000 × g for 30 min, the resulting supernatant was collected. Protein concentration was determined using a Bio-Rad protein assay (Bio-Rad, Hercules, CA). For perigonadal tissue, a GoldBio lysis buffer was used. Subsequently, 40 μg of protein was mixed with a 5-fold sample buffer, heated to boiling for 10 min, and subjected to electrophoresis on a 10% SDS-polyacrylamide gel (SDS-PAGE) at a constant current of less than 100 mA. The separated samples were then transferred onto PVDF membranes (Millipore Corp., Bedford, MA). After blocking with 1% bovine serum albumin in a 20 mM Tris−HCl buffer for 1 h at room temperature, immunoblotting was performed using primary antibodies directed at target and control proteins. Horseradish peroxidase-conjugated secondary antibodies were applied to the blots for detection, and the expression of the target proteins was assessed and quantified using ImageJ software.

### 2.8 Short-Chain Fatty Acid (SCFA) Analysis

Short-chain fatty acid (SCFA) content analysis was meticulously conducted through a series of steps involving organic solvent extraction and gas chromatography−mass spectrometry (GC-MS).^25^ Ethyl acetate was utilized as the extraction solvent to separate SCFAs from fecal fats and proteins. Mouse cecal feces (0.1 g) were mixed with 1 mL of 0.5% phosphoric acid and homogenized. After centrifugation, 0.75 mL of ethyl acetate was added, followed by additional homogenization. Subsequent centrifugation at 4 °C and 18,000 × g for 10 min yielded 200 μL of supernatant, which was combined with 800 μL of 625 μM 4-methylvaleric acid as an internal standard and filtered through a microfilter. GC-MS analysis was performed using an Agilent Technologies 7890 Gas Chromatograph System with a 5975 inert Mass Selective Detector and a DB-WAXetr capillary column. Fixed flow rate was 1 mL/min, the injection volume was 1 μL, and specific temperature settings were applied for the injector, auxiliary heater (Aux), ion source, and quadrupole. Detection mass ranged from 30 to 250 *m*/*z*, with a solvent delay of 3.5 min. A meticulously programmed temperature gradient for the oven started from 90 °C, gradually increasing to 150 °C over the first 4 min, then to 170 °C over the next 4 min, followed by a ramp to 250 °C in the subsequent 4 min, and finally holding at 250 °C for the last 2 min of the run. This specific gradient was designed to efficiently separate and accurately quantify the SCFAs in the samples. The peak areas were integrated and compared against the internal standard curve, allowing for precise quantification of the SCFA concentrations within the samples.^26^

### 2.9 Microbial Analysis

The PCR primer sequences, as previously reported,^27^ were utilized to amplify the variable region of the 16S rRNA gene, and the PCR conditions were adapted from previous studies.^28,29^ Subsequently, the amplicons were employed to construct index-labeled libraries using the Illumina DNA Library Preparation (Illumina, San Diego). The Illumina MiniSeq NGS System (Illumina) was employed to conduct paired-end sequencing (2 × 150 bp), generating more than 100,000 reads.^30^ A metagenomics workflow was utilized to classify organisms based on the 16S rRNA data, utilizing the Greengenes database (https://greengenes.lbl.gov/).^31^ The workflow provided taxonomic classifications at multiple levels, including kingdom, phylum, class, order, family, genus, and species, as the output.^32^

### 2.10 Statistical Analysis

The statistical evaluations were accomplished with one-way analysis of variance (ANOVA), followed by Duncan’s multiple range test. Statistical analyses were performed using SPSS software (Version 26.0.0). The results were considered to be significant for *p* ≤ 0.05. All data were presented as the means ± SEM.

## 3 Results

### 3.1 Effects of LR069 and LB031 on Body Weight, Food Intake, and Organ Weight in HFD-Fed Mice

**Figure 1:**
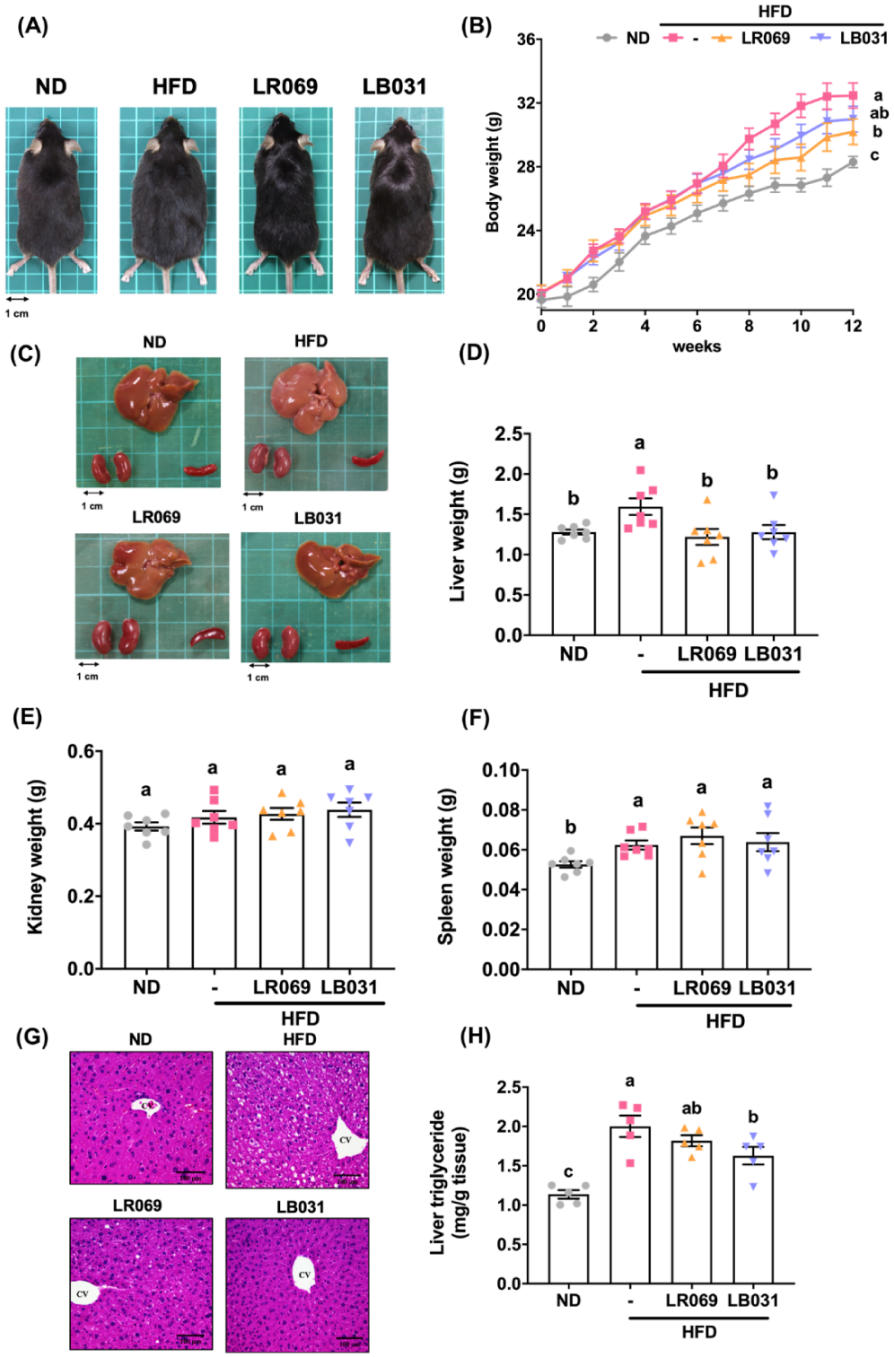
Effects of LR069 and LB031 on body weight, organs, H&E staining of liver, and liver triglyceride in HFD-fed mice. (A) Representative photographs of each group. (B) Weekly average body weight of each group, expressed as means ± SEM (C)−(F) Representative photographs of each group’s organs (liver, kidneys, and spleen) and organ weights. (G) Pathological assessment by H&E staining of the liver (200× magnification; length of the scale on the right = 100 μm). (H) Liver triglycerides. Data are expressed as the means ± SEM. Values with different letters (a−c) differ significantly (*p* < 0.05) among the compared groups.

After 12 weeks of a high-fat diet (HFD), mice in the HFD group exhibited a rounder body shape compared to the normal diet (ND) group. However, administration of probiotics LR069 and LB031 to the HFD group resulted in decreased body weight and a leaner appearance (Figure 1A). Table S1 displays the observed changes in body weight across the groups during the experiment. The HFD group showed a notably faster weight gain trend, with a 4.17 g difference compared to the ND group by the twelfth week, successfully inducing obesity in these mice. There was no significant difference in average body weight between the probiotic-fed groups, LR069 and LB031. By the eighth week, the LR069 group began to diverge from the HFD group, while the LB031 group maintained a slightly lower average body weight (Figure 1B). Before sacrifice at the twelfth week, the LR069 group had an average body weight of 30.20 ± 2.29 g, which was 2.27 g lower than the HFD group (32.47 ± 2.23 g), showing a significant difference (Figure 1B and Table S1). The HFD group exhibited the highest average body weight gain of 12.39 ± 2.12 g, while the LR069 group had a gain of 10.07 ± 2.01 g, 18.7% less than the HFD group. These results suggest that probiotic LR069 can mitigate the weight gain induced by a high-fat diet in mice. Average daily food intake did not significantly differ between groups, indicating that the effects of probiotics LR069 and LB031 on body weight were not due to appetite suppression or reduced energy intake (Table S1). Figure 1C displays the average weights of the mice’s essential organs. Notably, there were no significant differences in organ weights across the groups, suggesting that daily administration of LR069 and LB031 did not impact organ weight (Figure 1E,F).

### 3.2 Effects of LR069 and LB031 on Blood Biochemical Values in HFD-Fed Mice

Hyperlipidemia is an important risk factor for cardiovascular disease.^33^Table 1 shows serum AST/GOT, ALT/GPT, TG, TC, LDL, and fasting blood glucose. The results showed that liver function indicators AST/GOT and ALT/GPT values did not differ significantly among dietary groups, indicating that LR069 and LB031 did not induce body toxicity at the given dose. Additionally, the results showed that the HFD group was associated with increased TG, TC, and LDL levels compared with the ND group. However, administration of LR069 and LB031 significantly reduced TG levels. LB031 also reduced serum TC and LDL levels. Furthermore, fasting blood glucose values were significantly lower in the LR069 group compared with the HFD group. These results showed that LR069 and LB031 have the potential to improve dyslipidemia and prevent cardiovascular disease.

**Table 1:** Effect of LR069 and LB031 on Blood Biochemical Values in HFD-Fed Mice^i^

	ND	HFD	LR069	LB031
AST/GOT (U/L)	142.2 ± 15.98^a^	189.6 ± 74.3^a^	172.8 ± 93.3^a^	195.5 ± 107.2^a^
ALT/GPT (U/L)	27.07 ± 3.87^a^	33.39 ± 9.04^a^	26.94 ± 5.87^a^	26.47 ± 4.64^a^
TG (mg/dL)	49.75 ± 22.66^ab^	59.88 ± 9.71^a^	30.59 ± 16.30^b^	34.35 ± 22.53^b^
TC (mg/dL)	73.73 ± 7.01^c^	156.50 ± 19.45^a^	136.98 ± 32.40^ab^	131.66 ± 22.87^b^
LDL (mg/dL)	7.20 ± 1.63^c^	34.88 ± 8.36^a^	26.40 ± 13.78^ab^	23.59 ± 7.60^b^
fasting blood glucose (mg/dL)	115.5 ± 4.9^c^	155.6 ± 18.2^a^	132.0 ± 11.6^b^	145.9 ± 11.4^a^

i Data are expressed as mean ± SEM. The significance of difference among the four groups was analyzed by one-way ANOVA and Duncan’s multiple range tests. The values with different letters (a−c) are significantly different (*p* < 0.05) between each group.

### 3.3 Effects of LR069 and LB031 on Liver and Adipose Tissue in HFD-Fed Mice

As shown by liver appearance and weight and serum biochemical values, administration of LR069 and LB031 can slow lipid accumulation in the liver caused by high-fat diet. Figure 1G,H show differences in liver color and triglycerides between the HFD group and the ND group. It is speculated that long-term high-fat diet will cause fatty infiltration in the liver.^34^ H&E staining of liver sections showed obvious white vacuoles in the HFD group, indicating lipid accumulation in the liver of the HFD group. Lipids from macrovascular steatosis accumulate in hepatocytes due to increased triglyceride synthesis.^35^ Administration of LR069 and LB031 significantly improved the liver fat accumulation caused by high-fat diet.

**Figure 2:**
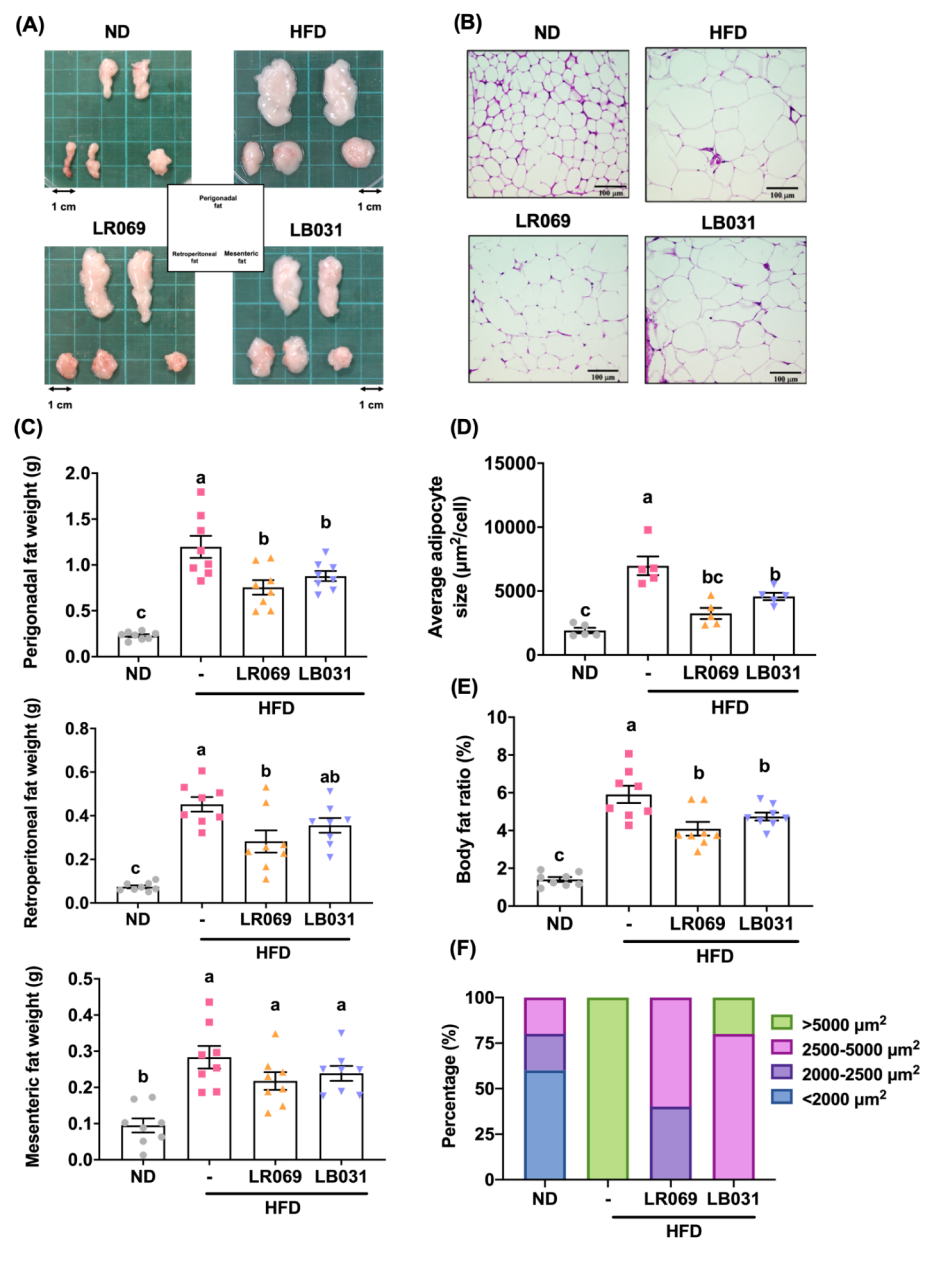
Effect of LR069 and LB031 on adipose tissue in HFD-fed mice. (A) Representative appearances of perigonadal, mesenteric, and retroperitoneal tissues. (B) Representative image of H&E-stained perigonadal adipose tissue (200× magnification; length of the scale on the right = 100 μm). (C) Adipose tissue weights. (D) Average adipocyte size. (E) Body fat ratio (%) is calculated as the total weight of adipose tissues/body weight ×100. (F) Percentage of the adipocyte size distribution of perigonadal adipose tissue. Data are expressed as the means ± SEM. Values with different letters (a−c) differ significantly (*p* < 0.05) among the compared groups.

The visceral fat of mice can be mainly divided into gonadal (perigonadal/epididymal), peritoneal (retroperitoneal), and mesenteric (mesenteric) fat. Both appearance and weight of all of these tissues were higher in the HFD group than in the ND group, and the fat volume of the LR069 and LB031 groups was slightly smaller than that of the HFD group (Figure 2A). As shown in Figure 2C, LR069 and LB031 supplements could reduce the weight of adipose tissue, compared with the HFD group, and the effect on gonadal (perigonadal/epididymal) fat was the most significant (p *<* 0.05). Probiotic LR069 also reduced the weight of peritoneal fat (*p* < 0.05), with a slightly better effect than that of probiotic LB031. After the experimental mice were sacrificed, the gonadal fat was sectioned and stained (H&E). At a magnification of 200x, the adipocytes in the HFD group were similar to those in the LB031 group. The specific area was significantly larger, and the size of adipocytes in the LR069 group tended to be smaller compared with the HFD group (Figure 2B,D). Taken together, the above results indicated that probiotic LR069 has the ability to inhibit visceral fat accumulation.

### 3.4 Effect of LR069 and LB031 on Lipid Metabolism in HFD-Fed Mice

**Figure 3:**
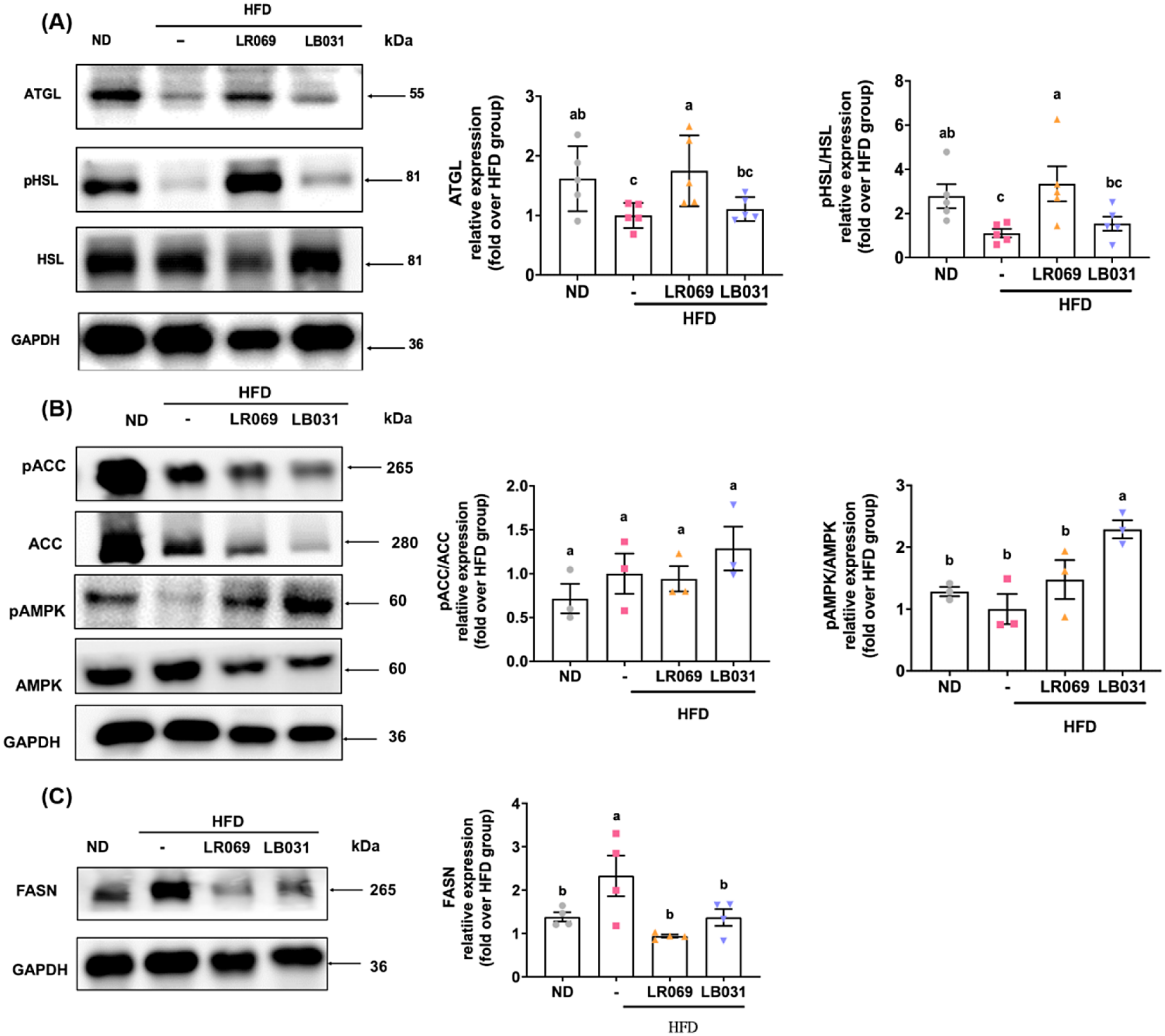
Effect of LR069 and LB031 on lipid metabolism-related proteins in the perigonadal adipose tissue in HFD-fed mice. (A) Representative Western blot images of ATGL, p-HSL, HSL, and their quantification, using GAPDH as internal control. (B) Representative Western blot images of p-ACC, ACC, p-AMPK, AMPK, and their quantification, using GAPDH as internal control. (C) Relative Western blot images of FASN and its quantification, using GAPDH as internal control. The protein bands were quantified using ImageJ software. Data are expressed as the means ± SEM. Values with different letters (a−c) differ significantly (*p* < 0.05) among the compared groups.

Adipose triglyceride lipase (ATGL) and hormone-sensitive lipase (HSL) constitute pivotal enzymes in the intracellular catabolism of triacylglycerols.^36^ Dysregulation of lipogenesis and lipolysis can lead to fat accumulation and eventual obesity.^37^ This study investigated the effects of LR069 and LB031 on the expression of lipolysis-related proteins, specifically ATGL and HSL. As shown in Figure 3A, the experimental results revealed that the protein expression of ATGL in adipose tissue increased significantly after administration of LR069. In addition, analysis of p-HSL/HSL protein expression showed that compared with the HFD group, LR069 supplementation promotes HSL phosphorylation and thus improve lipid metabolism in adipose tissue (Figure 3A). Elevated de novo lipogenesis and diminished fatty acid oxidation likely contribute to the accumulation of adipose tissue in obesity.^38^Figure 3B shows that LB031 supplementation significantly phosphorylated AMPK. The LR069 showed a marginal increase in the levels of the p-AMPK/AMPK. AMPK negatively regulates ACC, resulting in a decrease in malonyl-CoA concentration, leading to a subsequent reduction in fatty acid synthesis.^39^ As shown in Figure 3B, there was an observable rise in the expression of the p-ACC/ACC in the LB031 group. Furthermore, FASN is the transcription factor for fatty acid synthase, and Figure 3C showed that supplementation of LR069 and LB031 significantly reduced FASN expression. These findings indicate that probiotics supplementation can increase lipid breakdown and potentially improve lipid metabolism in the perigonadal tissue.

### 3.5 LR069 and LB031 Improved Inflammation in HFD-Fed Mice

Obesity is characterized by a state of chronic, low-grade inflammation.^40^ Obesity induces the generation of various pro-inflammatory factors, including some adipokines, leading to a state of chronic inflammation. Adipokines encompass a range of pro-inflammatory and anti-inflammatory factors; the majority of the pro-inflammatory adipokines exhibit elevated levels in obesity.^41^

**Figure 4:**
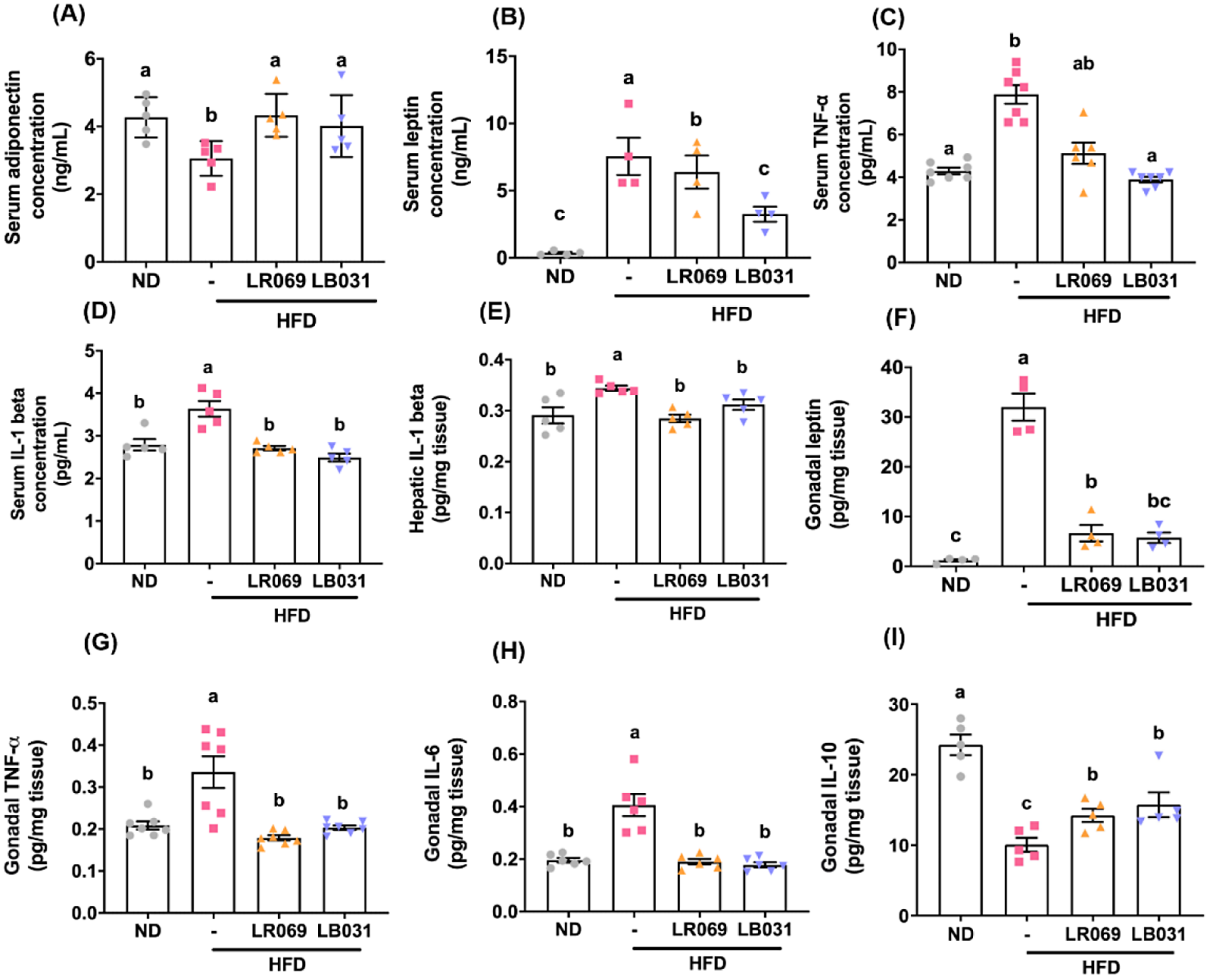
Effect of LR069 and LB031 on cytokines in HFD-fed mice. (A) Serum adiponectin concentration. (B) Serum leptin concentration. (C) Serum TNF alpha concentration. (D) Serum IL-1 beta concentration. (E) Hepatic IL-1 beta. (F) Gonadal leptin. (G) Gonadal TNF-alpha. (H) Gonadal IL-6. (I) Gonadal IL-10. Data are expressed as the means ± SEM. Values with different letters (a−c) differ significantly (*p* < 0.05) among the compared groups.

In the initial phase of this study, we focused on two key adipokines: leptin and adiponectin (ADIPOQ). The results, as depicted in Figure 4A,B, demonstrated a statistically significant increase in serum ADIPOQ levels and a decrease in serum leptin levels in groups treated with LR069 and LB031 on a high-fat diet (HFD). Additionally, Figure 4F illustrates that LR069 and LB031 were effective in reducing leptin content in gonadal tissues. IL-1β was measured in both serum and liver tissues due to its critical involvement in nonalcoholic fatty liver disease (NAFLD), a condition prevalent in obesity and type 2 diabetes. IL-1β is known to promote hepatic steatosis by enhancing lipogenic pathways, particularly through the upregulation of fatty acid synthase, essential for triglyceride synthesis in the liver.^42,43^ Correspondingly, cytokine analysis indicated a reduction in IL-1β levels in both serum (Figure 4D) and hepatic tissues (Figure 4E) in the LR069 and LB031 treatment groups. Furthermore, IL-6 and TNF-α were quantified in adipose tissue, reflecting their secretion by adipocytes and their correlation with body fat mass and distribution. These cytokines are integral to inflammatory responses and are implicated in metabolic dysfunctions, such as insulin resistance.^44^ The administration of LR069 and LB031 was shown to reduce levels of TNF-α and IL-6 in gonadal tissues (Figure 4G,H), with TNF-α specifically measured in serum due to its significant role in systemic inflammation linked to insulin resistance and adipose tissue dysfunction in obesity.^45^ Results confirmed that probiotic treatment effectively reduced serum TNF-α expression (Figure 4C). Remarkably, there was also an elevation in the anti-inflammatory cytokine IL-10 in the gonadal tissues of the LR069 and LB031 groups (Figure 4I), underscoring a comprehensive modulation of inflammation. This finding highlights the potential therapeutic benefits of these *Lactobacillus* strains in mitigating metabolic inflammation, further supported by their impact on adipokines and cytokines across various tissues.

**Figure 5:**
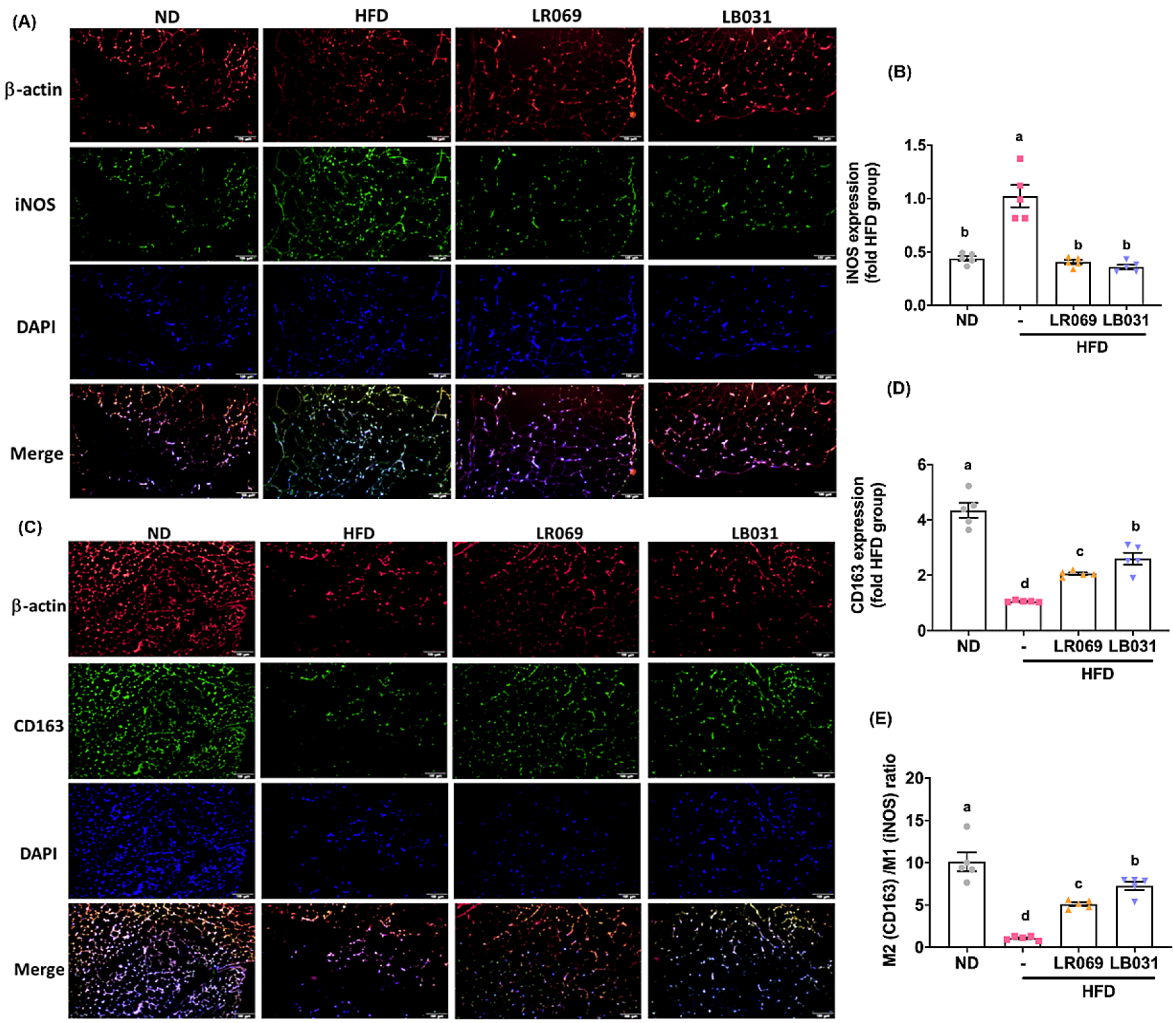
LR069 and LB031 regulate M1/M2 adipose tissue macrophages (ATMs) in perigonadal adipose tissue. (A) Immunofluorescence staining of β-actin (red), M1 marker iNOS (green), and nuclei (DAPI, blue) (200× magnification; length of the scale on the right = 100 μm). (C) Immunofluorescence staining of nuclei (blue), β-actin (red), and M2 marker CD163 (green) (200× magnification; length of the scale on the right = 100 μm). (B, D) Quantification of iNOS and CD163. (E) M2 (CD163)/M1 (iNOS) ratio. The immunofluorescence staining was quantified using ImageJ software. Data are expressed as the means ± SEM. Values with different letters (a−d) differ significantly (*p* < 0.05) among the compared groups.

In addition to cytokine profiles, our study focused on macrophage polarization within perigonadal adipose tissue, using iNOS and CD163 as markers for M1 and M2 macrophages, respectively. iNOS and CD163 are commonly used markers for delineating mouse M1/M2 macrophage polarization. Investigating the expression of iNOS (M1) and CD163 (M2) within perigonadal adipose tissue, particularly through the application of immunofluorescent staining techniques, enables a nuanced understanding of M1/M2 adipose tissue macrophages (ATMs).^46,47^ The selection of iNOS as an M1 marker is based on its established role as a pro-inflammatory enzyme, predominantly expressed in classically activated M1 macrophages, and its involvement in the pathogenesis of inflammation and fibrosis.^48−50^ The expression of iNOS is indicative of a pro-inflammatory state and has been implicated in the metabolic disturbances associated with obesity.^51^ Conversely, CD163 was selected as the M2 marker due to its association with the anti-inflammatory activities of alternatively activated M2 macrophages. As illustrated in Figure 5A−D, immunofluorescent staining indicates that, relative to the HFD group, the LR069 and LB031 groups exhibit a notable reduction in iNOS production and an increased expression of CD163, with LB031 displaying the most pronounced effect on CD163. Calculating the M2/M1 ratio further reveals a statistically significant difference between the sample and the induction group, as depicted in Figure 5E, with LB031 exhibiting the most conspicuous impact.

### 3.6 Effect of LR069 and LB031 Changed the Gut Microbiota Composition in HFD-Fed Mice

Many studies have pointed out that the presence of microbial communities in the intestine may affect the energy intake from the diet, thereby affecting the degree of obesity of the host.^52^ Therefore, this experiment further explores the impact of obesity on the intestinal bacterial phase, showing that LR069 and LB031 affect the intestinal flora.

The computations of Good’s coverage index, Shannon index, and Simpson’s index did not show significantly different bacterial community richness and diversity between the HFD group and other studied groups (Figure S1A−C). However, according to the results in Figure S1D,E, the Venn diagram and UpSet plot can present the number of common or unique OTUs between each group. Hence, it can be deduced that HFD associated with LR069 or LB031 supplements will produce changes in gut bacteria. To study the similarities between different samples, a cluster tree can also be constructed by cluster analysis on the samples. In environmental biology, UPGMA (unweighted paired-group method using arithmetic means) is a commonly used cluster analysis method.^53^ According to Figure S1F, the HFD group is significantly different from the other three groups, and the bacterial flora after administration of LR069 and LB031 is similar to that of the ND group. Therefore, the administration of LR069 or LB031 to HFD-fed mice has a significant impact on the composition of intestinal bacterial flora and can regulate the imbalance of intestinal bacteria induced by HFD.

### 3.7 Effect of LR069 and LB031 on the Gut Microbiota of Mice with High-Fat-Diet-Induced Obesity

**Figure 6:**
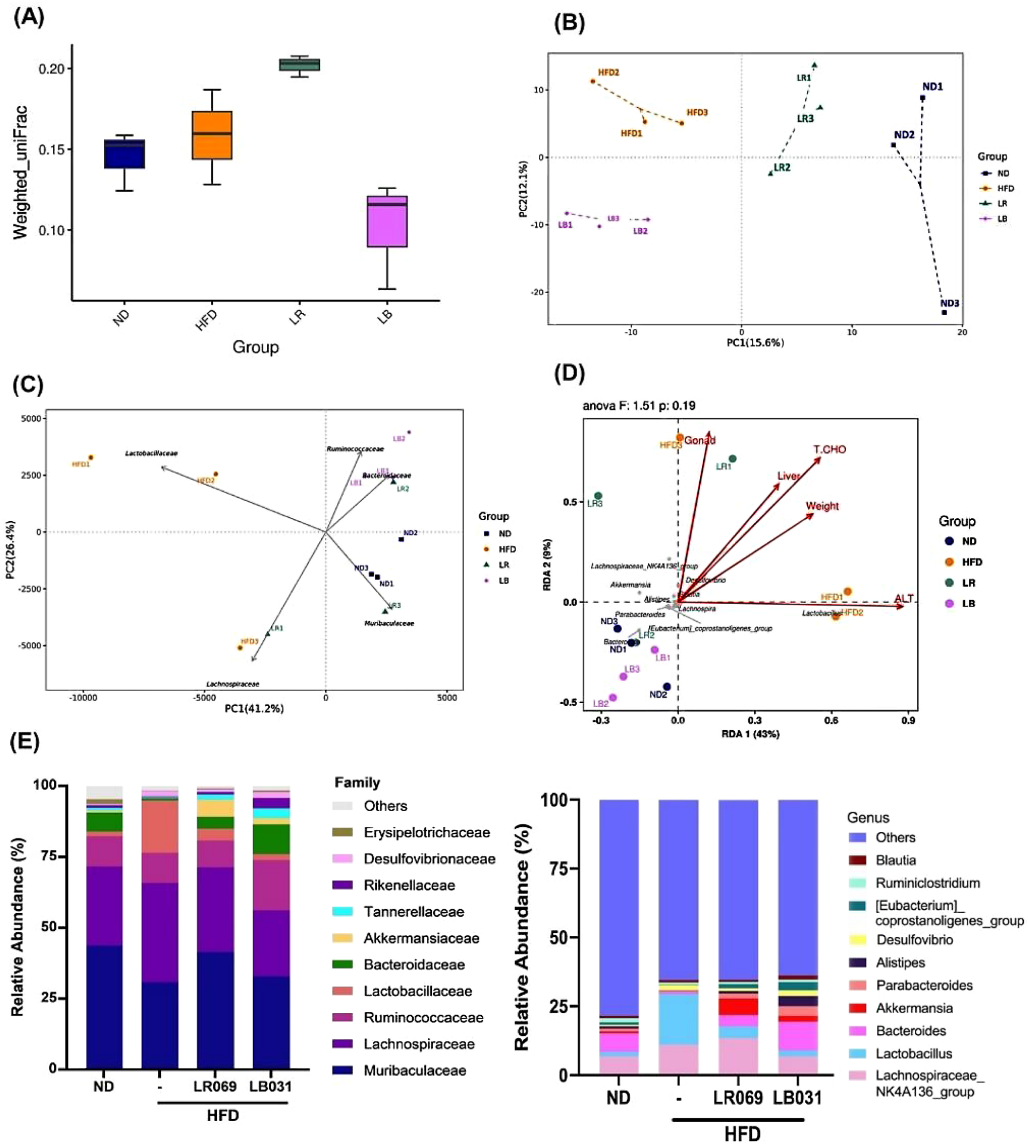
Effect of LR069 and LB031, changing the gut microbiota composition in HFD-fed mice. (A) Weighted UniFrac values show the difference in β-diversity. (B) PCoA. (C) PCA plots at family level. (D) RDA analysis. The chart of the correlation between environmental factors and genus level bacterial species between groups. (E) Family and genus classification. *n* = 3 for each analysis, LR = LR069, LB= LB031.

Weighted UniFrac values indicate dissimilarity in β-diversity, considering the relative abundance of microbial taxa across samples. Higher values suggest increased dissimilarity in overall microbial composition.^54^ As depicted in Figure 6A, the weighted UniFrac values distinctly illustrate differences in β-diversity. LR069 exhibits notably higher weighted UniFrac values, underscoring its unique impact on the dissimilarity of microbial communities.

By employing PCA analysis, variations in gut microbiota among samples in animal experiments can be elucidated. The resultant two-dimensional coordinate plot visually illustrates differences in microbiota composition.^55^ Shorter distances in the plot indicate a higher degree of similarity in gut microbiota composition among the samples in the animal study. According to the principal coordinates analysis (PCoA) results in Figure 6B, mice subjected to HFD exhibited a distinct distribution compared to those in the ND group, and different distributions were also observed after the administration of LR069 and LB031.

When conducting PCA analysis using a covariance matrix, the dominant variables influencing the data are evaluated, and the five species with the most significant contributions (ASV) are depicted in PCA plots at the family level. In Figure 6C, the prominent bacterial species contributing the most are *Muribaculaceae*, *Bacteroidaceae*, *Ruminococcaceae*, *Lactobacillaceae*, and *Lachnospiraceae*. Notably, *Muribaculaceae* exhibits a strong association with the ND and LR069 groups, while *Bacteroidaceae* and *Ruminococcaceae* are linked to the LB031 group, and *Lactobacillaceae* shows a high correlation with the HFD group. Constrained ordination, a technique merging correspondence analysis and multiple regression, includes environmental factors in each step.^56^ Also termed multivariate direct gradient analysis, it investigates the relationship between species and environmental factors, pinpointing significant drivers influencing sample distribution. Redundancy analysis (RDA) is a widely used method within this context.^57^ In Figure 6D, the impact of environmental factors on bacterial species is depicted by arrow lengths, while arrow angles represent correlations between factors. Acute angles indicate positive correlations, whereas obtuse angles signify negative correlations. Species, portrayed as gray dots, encompass those exhibiting negative correlations with environmental factors such as body weight, liver weight, ALT, total cholesterol in serum, and gonad weight. Notably, *Lachnospiraceae*_NK4A136_group, *Akkermansia*, *Alistipes*, *Parabacteroides*, and *Bacteroides* emerge among the top 10 contributors. The differences in intestinal bacterial composition among groups were analyzed at the bacterial family and genus levels, as depicted in Figure 6E. At the bacterial family hierarchy, the HFD group exhibited an increase in *Lactobacillaceae*, while the other groups demonstrated an elevation in *Tannerellaceae* and *Bacteroidaceae*. Following the administration of LR069 and LB031, there was an augmentation in *Akkermansiaceae*. Additionally, at the genus level, the LR069 and LB031 groups showed increased abundance of *[Eubacterium]_coprostanoligenes_*group, *Alistipes*, *Parabacteroides*, *Akkermansiaceae*, and *Bacteroides*. Administration of LR069 or LB031 effected several changes in abundance.

### 3.8 Effects of LR069 and LB031 on Gut Microbiome Functionality and SCFA Production in Mice with High-Fat-Diet-Induced Obesity

**Figure 7:**
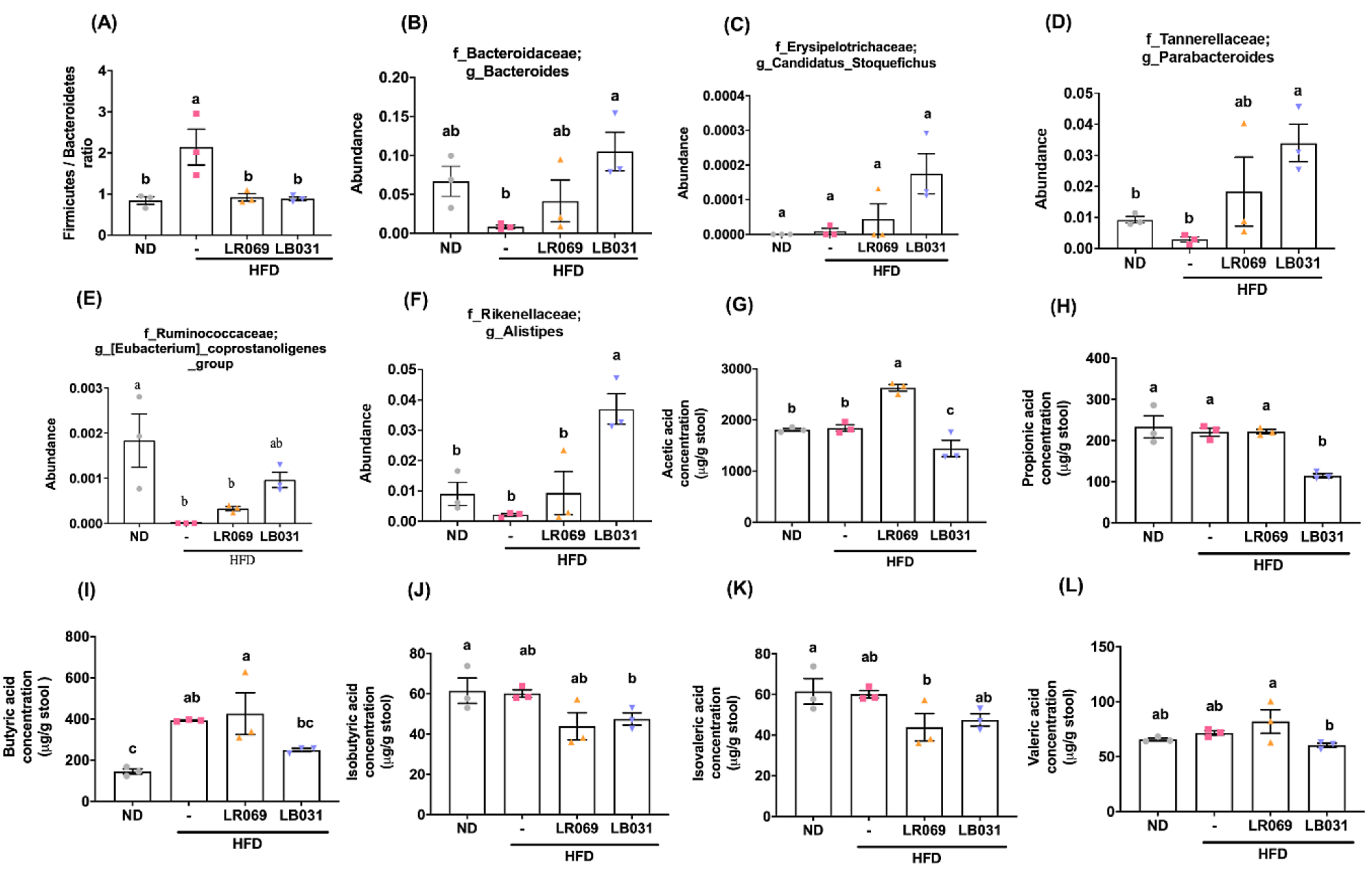
Comparison of gut microbiota and fecal SCFA levels among all experimental groups in HFD-fed mice. (A) *Firmicutes*/*Bacteroidetes* ratio. Genus changes in gut microbiota: (B) *Bacteroides*. (C) *Candidatus_Stoquefichus*. (D) *Parabacteroides*. (E) *[Eubacterium]_coprostanoigenes*. (F) *Alistipes*. (G) Acetic acid. (H) Propionic acid. (I) Butyric acid. (J) Isobutyric acid. (K) Isovaleric acid. (L) Valeric acid. Data are expressed as the means ± SEM. Values with different letters (a−c) differ significantly (*p* < 0.05) among the compared groups.

Many previous studies have pointed out that a high-fat diet will increase the ratio (F/B ratio) of the two major bacterial phyla Firmicutes and Bacteroidetes in the intestine, leading to bacterial imbalance.^58,59^ The HFD group exhibited the highest F/B ratio among the groups, as demonstrated in Figure 7A. Conversely, treatment with LR069 and LB031 probiotics resulted in an increased number of *Bacteroidetes*, thus lowering the F/B ratio, as shown in Figure 7B. This adjustment suggests a restoration toward a more balanced and healthier gut microbiota. At the genus level, we observed an increased abundance of *Candidatus_Stoquefichus*, *Parabacteroides*, and *[Eubacterium]_coprostanoligenes_*group in the LR069 and LB031 treated groups (Figures 7C-7E), indicative of the probiotics’ impact on promoting beneficial microbial communities. Notably, the LR031 group displayed a significant rise in the abundance of *Alistipes* (Figure 7F), a genus associated with positive gut health outcomes.

The influence of the gut microbiota extends beyond composition to functional metabolic activities, particularly the production of short-chain fatty acids (SCFAs). These crucial metabolites, generated through the bacterial fermentation of nondigestible carbohydrates in the colonic lumen, serve as a primary energy source for colonocytes and play a vital role in host metabolism.^60^ Our analysis using gas chromatography−mass spectrometry (GC-MS) revealed that while most SCFA levels remained stable in the HFD group, LR069 administration led to a significant increase in fecal acetic acid concentrations (Figure 7G). Additionally, trends toward increased butyric and valeric acid levels were observed, underscoring the probiotics’ role in enhancing beneficial metabolic functions.

In summary, the alterations in the gut microbiota composition and functionality induced by LR069 and LB031, particularly the reduction in the F/B ratio and the increase in beneficial SCFA production, reflect the potential of these probiotics to counteract the negative effects of a high-fat diet on the gut ecosystem. These changes signify a move toward a healthier gut environment, with implications for improving overall host metabolic health.

## 4 Discussion

Recent research has increasingly recognized the role of probiotics in addressing metabolic disorders, inflammation, and obesity-related weight gain, as evidenced in both animal and human studies.^61,62^ Our findings with *Lactobacillus rhamnosus* SG069 (LR069) and *Lactobacillus brevis* SG031 (LB031) complement this body of knowledge, showing significant metabolic improvements in mice on a high-fat diet (HFD). Similar to the study on *Lactobacillus rhamnosus* GG, which highlighted the strain’s ability to prevent diet-induced insulin resistance,^63^ LR069 and LB031 demonstrated substantial reductions in body weight, adipose tissue mass, and liver weight, showcasing their potential in countering obesity’s adverse effects.

The histological examination in our study aligns with findings from *Lactobacillus rhamnosus* BST-L.601 research, where reduced adipogenesis and lipogenesis were observed.^64^ Our detailed investigation into the metabolic pathways revealed that LR069 enhanced lipolysis, evidenced by increased ATGL expression and HSL phosphorylation, while LB031 promoted fatty acid oxidation, as indicated by AMPK phosphorylation, paralleling the metabolic improvements seen with *Lactobacillus brevis* strain NJ42 in reducing weight gain and improving glucose tolerance.^65^

Moreover, our study’s observations of improved lipid metabolism and reduced inflammatory markers, such as increased ADIPOQ levels and decreased TNF-alpha, leptin, and IL-1 beta, underscore the therapeutic potential of the probiotic strains LR069 and LB031. Our findings align with existing literature on the benefits of probiotics in reducing inflammation and modulating gut microbiota, similar to interventions using *Lactobacillus rhamnosus* LS-8 and *Lactobacillus crustaceans* MN047.^66^ However, our specific focus on the M2 macrophage phenotype and IL10 in adipocytes highlights key and often underexplored mechanisms by which these probiotics exert their effects, highlighting their role in promoting an anti-inflammatory state within adipose tissue. Our research not only corroborates the documented benefits of *Lactobacillus* strains in metabolic health but also significantly advances this knowledge by elucidating the specific contributions of LR069 and LB031. Through the investigation, we’ve detailed how these strains impact lipid metabolism regulation and immune response modulation, providing a comprehensive view of their roles in metabolic health. The study’s detailed mechanistic insights enhance our understanding of probiotics’ potential, positioning LR069 and LB031 as promising agents for therapeutic interventions against obesity and related metabolic disorders. Further, our exploration of gut microbiota composition revealed significant alterations induced by these strains, underscoring their ability to modulate the microbiome and improve metabolic health, resonate with the outcomes from heat-killed *Lactobacillus brevis* KB290, showing reduced visceral fat accumulation and modulated gut microbiota in mice.^67^

Notable examples include the administration of *Bifidobacterium pseudocatenulatum* CECT 7765, which exhibited significant anti-inflammatory properties in obese mice subjected to a high-fat diet.^68^ The probiotic formulation VSL#3, comprising *Lactobacillus* and *Bifidobacteria* strains, showed therapeutic potential for enhancing insulin sensitivity and suppressing body weight gain in both mouse models and human trials.^69,70^ The F/B ratio has been proposed as a potential biomarker for obesity, suggesting that a higher abundance of the Bacteroidetes phylum may contribute to preventing weight gain.^71^ Studies have identified microbial biomarkers associated with health, including the family *Eubacterium_coprostanoligenes*, *Tannerellaceae*, and genera such as *Lachnospiraceae* NK4A136 group, *Parabacteroides*, and *Akkermansia*.^72^ Genera like *Bacteroides*, *Akkermansia*, and *Alistipes* are abundant in nonobese populations and inversely associated with obesity and type 2 diabetes.^61,73^ In studies of chronic jet lag, *Candidatus _Stoquefichus* was found in greater abundance in healthy mice. The presence of *Candidatus_Stoquefichus* has been mentioned in healthy subjects compared to patients with Parkinson’s disease but has not been widely discussed in recent studies.^74,75^ The increase in the abundance of specific taxa, such as *Candidatus_Stoquefichus*, *[Eubacterium]_coprostanoligenes_*group, *Parabacteroides*, and *Alistipes*, underlines the potential of LR069 and LB031 in reshaping the gut microbiome to favor metabolic health. Literature related to gut microbiota indicates that *L. plantarum* Dad-13 altered gut microbiota composition, resulting in reduced body weight and BMI in a double-blind, placebo-controlled trial.^62^ LR069 supplementation resulted in a significant increase in acetate levels in mouse feces, indicating a potential role in regulating microbial fermentation processes to favor the production of beneficial short-chain fatty acids. This aligns with existing literature emphasizing the positive metabolic effects of increased acetate production in the gut. However, no significant changes in acetate levels were observed in the LB031 group, possibly due to variability or the specific effects of *Lactobacillus brevis*. In conclusion, LR069 and LB031 demonstrated robust antiobesity effects through multiple mechanisms, including modulation of lipid metabolism, anti-inflammatory responses, and alterations in gut microbiota composition. These findings contribute to a deeper understanding of the intricate interactions between probiotics, the gut microbiome, and host metabolism. Further investigations, particularly with a focus on long-term effects and signaling pathways, are warranted to fully elucidate the therapeutic potential of LR069 and LB031 in addressing obesity and related metabolic disorders.

## 5 Conclusion

In conclusion, our investigation into the strain-specific effects of *Lactobacillus rhamnosus* SG069 (LR069) and *Lactobacillus brevis* SG031 (LB031) in HFD-fed mice revealed distinct and significant impacts on metabolic parameters. LR069 demonstrated remarkable efficacy in combating obesity, as evidenced by substantial weight loss and enhanced adipose tissue lipolysis. The concurrent increase in *Akkermansia* and acetic acid suggests potential contributors to LR069’s antiobesity effects, possibly through modulation of gut microbiota composition and overall metabolism. Conversely, LB031 exhibited pronounced anti-inflammatory properties, marked by reductions in pro-inflammatory markers and an increase in the anti-inflammatory adiponectin. Although LB031 did not affect body weight significantly, it displayed potential in modulating lipid metabolic pathways, as evidenced by altered phosphorylation of key proteins. The highest M2/M1 ratio observed in adipose tissue further supports LB031’s anti-inflammatory role. These findings underscore the strain-specific nature of probiotic interventions, emphasizing the unique contributions of LR069 and LB031 to metabolic regulation in the context of a high-fat diet. The study highlights the potential therapeutic applications of these specific probiotic strains and suggests avenues for further research to elucidate the underlying mechanisms. These insights contribute to the development of targeted probiotic strategies for improving metabolic health in the context of obesity.

## Supporting Information Available

Detailed data on the effects of LR069 and LB031 on body weight changes and food intake in HFD-fed mice. Additionally, it includes analyses of alterations in the gut microbiota composition in HFD-fed mice treated with LR069 and LB031 (PDF)

## Abbreviations

ADIPOQ,: adiponectin
ALT,: alanine aminotransferase
AMPK,: AMP-activated protein kinase
ACC,: acetyl-CoA carboxylase
ATMs,: adipose tissue macrophages
AST,: aspartate aminotransferase
ATGL,: adipose triglyceride lipase
ELISA,: enzyme-linked immunosorbent assay
FASN,: Fatty acid synthase gene
GAPDH,: glyceraldehyde 3-phosphate dehydrogenase
H&E,: hematoxylin and eosin
HDL,: high-density lipoprotein cholesterol
HFD,: high-fat diet
HSL,: hormone-sensitive lipase
IL,: interleukin
LDL,: low-density lipoprotein
LR069,: *Lactobacillus rhamnosus* SG069
LB031,: *Lactobacillus brevis* SG031
ND,: normal diet
OTU,: Operational Taxonomic Unit
PVDF,: polyvinylidene difluoride
SCFA,: short-chain fatty acid
GC-MS,: gas chromatography−mass spectrometry
TC,: total cholesterol
TG,: triacylglycerol
TNF-α,: tumor necrosis factor-α
WAT,: white adipose tissue
