# Supplementary material for: Lactobacillus rhamnosus 069 and Lactobacillus brevis 031: Unraveling Strain-Specific Pathways for Modulating Lipid Metabolism and Attenuating High-Fat-Diet-Induced Obesity in Mice
Source: ACS Omega. 2024 May 16;9(26):28520–33. doi: 10.1021/acsomega.4c02514 (PMC11223209; doi:10.1021/acsomega.4c02514)
Supplement: Supplementary file 1 — ao4c02514_si_001.pdf [file ao4c02514_si_001.pdf]

Lactobacillus rhamnosus 069 and _Lactobacillus brevis_ 031: Unraveling Strain-Specific Pathways for Modulating Lipid Metabolism and Attenuating High-Fat Diet induced Obesity in Mice

Pin-Yu Ho[§], Ya-Chun Chou[§], Yen-Chun Koh[§,], Wei-Sheng Lin[§,†], Wei-Jen Chen[)], Ai-Lun Tseng[)], Chiau-Ling Gung[)], Yu-Shan Wei[)], and Min-Hsiung Pan [§,£,¶,*]

[§] Institute of Food Science and Technology, National Taiwan University, No. 1, Sec. 4, Roosevelt Road, Taipei 10617, Taiwan, ROC

[†] Department of Food Science, National Quemoy University, Quemoy County 89250, Taiwan, ROC

[)] Syngen Biotech Co., Ltd., Building A, No. 154, Kaiyuan Rd., Sinying, Tainan 73055, Taiwan

[£] Department of Public Health, China Medical University, 91, Hsueh-Shih Road, Taichung 40402, Taiwan, ROC

[¶] Department of Food Nutrition and Health Biotechnology, Asia University, 500, Lioufeng Rd., Wufeng, Taichung 41354, Taiwan, ROC

*** Please send all correspondence to:**

Dr. Min-Hsiung Pan

Institute of Food Science and Technology,

National Taiwan University,

No. 1, Section 4, Roosevelt Road, Taipei 10617, Taiwan.

Tel. no. +886-2-33664133

Fax. no. +886-2-33661771

E-mail: mhpan@ntu.edu.tw

Table S1. Effect of LR069 and LB031 on initial body weight, final body weight, body weight gain, and food intake in HFD-fed mice.

	ND	HFD	LR069	LB031
Initial body weight (g)	19.64 ± 1.34^{a}	20.08 ± 0.63^{a}	20.13 ± 1.26^{a}	20.05 ± 1.37^{a}
Final body weight (g)	28.30 ± 0.99^{c}	32.47 ± 2.23^{a}	30.20 ± 2.29^{b}	30.99 ± 2.15^{ab}
Body weight gain (g)	8.66 ± 1.35^{c}	12.39 ± 2.12^{a}	10.07 ± 2.01^{bc}	11.01 ± 1.73^{ab}
Food intake (g/day)	3.02 ± 0.44^{a}	2.94 ± 0.25^{a}	3.09 ± 0.42^{a}	3.08 ± 0.40^{a}

Data are expressed as mean ± SEM. The significance of difference among four groups was analyzed by one-way ANOVA and Duncan's multiple range tests. The values with different letters (a-c) are significantly different ($p < 0.05$) between each group.

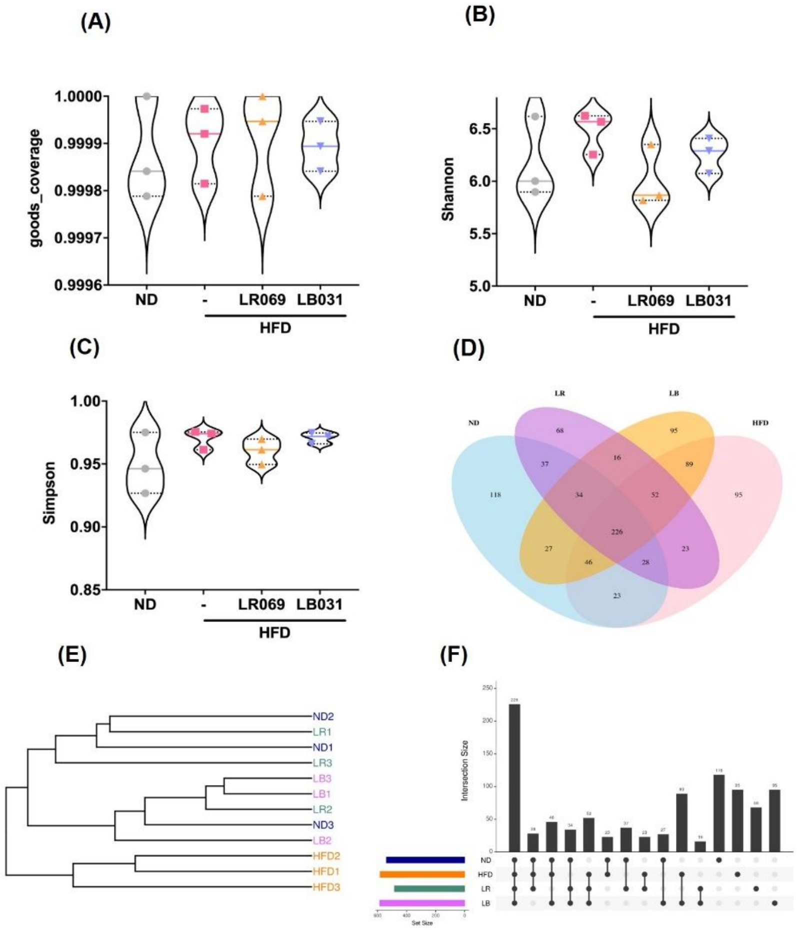

Figure S1. Effect of LR069 and LB031 changed the gut microbiota composition in HFD-fed mice. (A) goods_coverage. (B) Simpson. (C) Shannon. (D) Venn diagram. (E) UpSet plot. (F) UPGMA. (Unw eighted Paired-Group Method Using Arithmetic Means). n= 3 for each analysis, LR=LR069, LB= LB031.
